# Coping with Covid-19: The case of the National Library of Jamaica

**DOI:** 10.1177/0955749020984937

**Published:** 2020-08

**Authors:** Beverley Lashley, Nicholas Graham, Nicole Prawl

**Affiliations:** National Librarian, 128531 National Library of Jamaica, Kingston, Jamaica; User Services and Conservation, 128531 National Library of Jamaica, Kingston, Jamaica; Preservation and Conservation Branch, 128531 National Library of Jamaica, Kingston, Jamaica

**Keywords:** business continuity plan, Covid-19, National Library of Jamaica, pandemics

## Abstract

While Asian and European countries were grappling with the Covid-19 pandemic, the first imported case from the United Kingdom surfaced in Jamaica on 10 March 2020. The article will trace the steps taken by the National Library of Jamaica (NLJ) before and during the pandemic while improving the value of our cultural heritage to Jamaica and the wider diaspora. The NLJ celebrated 40 years of sterling contribution to the development of Jamaica from March 1979 to April 2020. It has been a leading voice in the region in disaster management and preparedness. Through its Preservation and Conservation Branch, and oversight from the Disaster Preparedness Committee, the NLJ has become a training ground for professionals in the information and conservation field. While highlighting the work of the NLJ, the article will also present statements and surveys conducted on regional and international bodies. These organisations include the Association of Caribbean University, Research and Institutional Libraries (ACURIL), the Community of Directors of National Libraries (a subgroup of the International Federation of Library Associations (IFLA) and the Society of American Archivists. The surveys conducted gleaned a greater appreciation of the impact of Covid-19 on libraries and archives regionally and internationally. The article will address other pandemic crises that have affected Jamaica. It will also demonstrate that being proactive and prepared for viral episodes can mitigate the risks. The NLJ used the opportunity to facilitate service delivery while being socially distant from its patrons.

## The early days: Preservation and conservation at the National Library of Jamaica

The National Library of Jamaica (NLJ) is an agency of the Ministry of Culture, Gender, Entertainment and Sport with its mission to ‘enhance the quality of life and prosperity of all citizens by acquiring, preserving and providing access to Jamaica’s cultural and historical information through research and education’ (NLJ, 2020a). The NLJ has just concluded its celebration of 40 years (April 1979–March 2020) of sterling contribution to the development of Jamaica and has been a leading voice in the region in disaster management and preparedness. Through its Preservation and Conservation Branch and oversight from the Disaster Preparedness Committee, the NLJ has become a training ground for professionals in the information and conservation field. The Preservation and Conservation Branch of the NLJ preserves and cares the paper-based, leather and parchment materials in the library’s collection. The branch was established in the earlier 1980s and, at that time, concentrated mainly on bookbinding and not so much on conservation techniques. However, by the 1990s, this changed to incorporate conservation techniques such as map repairs and preservation techniques such as making individual archival boxes for books. The Branch facilitated prolonged life to badly deteriorated books by using acid-free papers, which helped to slow down the rate of deterioration along with the introduction of environmental factors such as proper storage, useful housekeeping techniques and a controlled climate environment. It should be noted that the West India Reference Library (WIRL), which was an arm of the Institute of Jamaica, was established in 1879 and became the base collection for the NLJ that was formed in 1979.

## Overview of outbreaks in Jamaica

The island of Jamaica has had its share of dealing with outbreaks, pandemics and disasters. Jamaica experienced an outbreak of the dreaded Asiatic cholera in 1850–1855. The disease was said to have spread over most of the island and claimed more than 31,000 lives (Senior, 1994).

The great earthquake of 1907 was a major catastrophe in Jamaica. The historians have captured the extent of this event, and resources in both photographs and written documentation are available at the NLJ. Still, little literature is available on the impact it had on libraries and their operations (Great Britain, Parliament, 1918). The Spanish flu lasted from the spring of 1918 to the early part of the summer of 1919. In the British Caribbean, as it was then called, it was reported that over 30,000 people were killed, many of them were Jamaicans, as Jamaica was said to be the first island that was affected (Gilcrest, 2020). The documented material focused more on the social impact due to pandemics rather than the impact on libraries (Historical Notes file on Diseases, Jamaica. n.d.)

The Severe Acute Respiratory Syndrome (SARS) said to be a form of the coronavirus did not have much of an impact on Jamaica. In some countries like Canada, measures were taken which involved quarantining persons, social distancing and the closing of institutions such as schools were mentioned. The impact of SARS on libraries has been detailed in the literature. The study reflects the research queries on health-related information and the response given by staff at the library (Harris et al., 2005).

The NLJ had encountered the dreaded mould beginning in the summer of 2012, which is still going on, though controlled. The first major disaster occurred on 22 April 2016, with flooding of the main building. The waterlogged items were successfully salvaged by the Preservation and Conservation Team. The onset of Covid-19 has presented a new challenge to the library, one that was never encountered before. So, the deliberations focused on the way forward in terms of the security and sanitisation of the national collection.

## A plan of action

A plan of action that included the input from staff was needed to determine the workplace protocols. These protocols would guide the actions of staff who would be interfacing with the public. The Manager, Research and Information Branch was charged with meeting with frontline staff to solicit suggestions/recommendations on what the library needed to put in place to ensure their safety. This branch interfaces primarily with the public. The meeting was held on 31 January 2020, and the recommendations were submitted to the National Librarian for her approval. The suggestions/recommendations included the:

- purchasing of wipes dispenser for clients to use to open the doors and wipe the keyboards;
- sanitisation stations at each contact point;
- hand sanitisers and masks at the front desk for clients;
- prohibit persons with flu-like symptoms from entering the building;
- sanitise contact areas at least every hour;
- ensure staff practice the sanitisation of their hands before leaving the building; and
- usual gloves and mask for clients and staff.

The NLJ had started mitigation measures to alleviate the risk to staff and clients through its Disaster Preparedness Committee before the first Covid-19 case was imported in Jamaica. On Monday, 9 March 2020, one day before Jamaica was to record its first case of Covid-19, a team comprising of all the managers from the service-oriented branches met to devise strategies to mitigate the risk to staff. The team comprises of Managers from Research and Information, Preservation and Conservation, Finance and Accounts, Maintenance and Property, and the Audio-Visual Technical Coordinator from the Audio-Visual and Micrographics Branch. In this meeting, suggestions/recommendations were made regarding the measures that could be put in place to ensure that the workplace remained sterile, and the risks of a staff member contracting the virus would be minimised. Below are the recommendations coming out of that meeting:

1. Purchase 6 month’s supply of the following items:
  - Face masks (staff and public);
  - Gloves (staff and public);
  - Sanitisers for all Branches;
  - Vinegar and peroxide to be purchased as a low-cost sanitisation method.
2. Bins for foyer and reading rooms to dispose the gloves and other cleaning agents
3. Bins for items used by external clients to be collected and sanitised by staff from the Preservation and Conservation Branch.
4. A hairdryer to be used for general sanitisation of some collections as a heat source.
5. Incubators to store collections after use as a means of sanitisation.
6. Additional dispensers for Cundall Room and the Preservation and Conservation Branch.
7. Heat box to be made to store cash collected by Finance and Accounts Branch to sanitise cash collected.
8. Wipes dispensers to be placed in bathrooms.
9. Lab coats to be purchased (staff serving public).
10. Signs to be placed at all sanitisers.
11. Investigate the use of hands-free doors for implementation.
12. General signage to advise the public regarding the new measures to be implemented.
13. Investigate the workplace protocol of interacting with clients who are exhibiting flu-like symptoms while using the collection/facility.
14. Investigate the public sector provisions as it relates to being absent from work as a result of contracting Covid-19.
15. Purchase infrared thermometers to test staff and clients when they enter the foyer.

Covid-19 has the NLJ team grappling on how to preserve the national collection for future generations. What should we do now in terms of accessibility? How do we protect ourselves and the public against Covid-19? How should we protect our collections against this invisible enemy?

Armed with these and other questions in mind, the User Services and Conservation Division of the NLJ met as a team on 10 March 2020 to discuss the way forward. This date is significant in the history of Jamaica as it marked the first imported Covid-19 positive case. The User Services and Conservation Division comprises of the following branches: Preservation and Conservation; Special Collections; Audiovisual and Micrographics; and Research and Information. The decisions made at the meeting as it relates to the care of the collection during Covid-19 were as follows:

1. Conduct research to ascertain what other libraries were doing.
2. Investigate the use of book incubators.
3. Place moveable garbage bins lined with bags on every floor that accommodates external clients. All returned items would be sent to the Preservation and Conservation Branch for sanitisation.
4. Disinfectant or sanitisers which have at least 70% alcohol is recommended by the World Health Organization (WHO). Alcohol has been used in our fight against mould and has proven to be effective. The outcome far outweighs the drawbacks, as alcohol tends to stain some of the older binding cloths.
5. The use of diluted bleach to wipe the metal shelves where books are stored was also a method that could be used on the shelves and not the books.
6. Placing the collection of books in the sun under a covered area is another method that can be implemented because ongoing research has indicated that heat is a recommended factor in the Covid-19 fight. Information coming out of a webinar facilitated by the Canadian Conservation Institute stated that, ‘A few observations are possible. In general, cool temperatures (4–6°C) prolong viral persistence while very warm temperatures (60°C and above) result in a rapid loss of virulence’ (Karsten, 2020). The purchase of a hairdryer was recommended to be used on the books as flu viruses are killed by heat above 75°C (Centers for Disease Control and Prevention (CDC), 2018). Jamaica is blessed with an abundance of sunshine, so these methods could be explored. The process would have to be monitored, and further investigations conducted.

As mentioned before, the first imported case from the United Kingdom surfaced in Jamaica on 10 March 2020 (Public Broadcasting Corporation of Jamaica, 2020a). At approximately 1:30 p.m., the Minister of Health and Wellness in a press conference announced that Jamaica had its first confirmed case of the novel coronavirus, now named as Covid-19 (Public Broadcasting Corporation of Jamaica, 2020b). The panic and mayhem in the country were widespread as several individuals flocked pharmacies and supermarkets to purchase hand sanitisers and other daily cleaning essentials. The earlier meetings at the NLJ from January onwards had resulted in the purchase of the items that were recommended. The NLJ had purchased and installed three-hand sanitiser dispensers, one at the front door entrance, one on the first floor and the other on the second floor. Signs were strategically positioned, advising external clients to sanitise their hands on entry and exit of the building. Staff was also encouraged to exhibit the same practice. Each branch was outfitted with hand sanitisers for frequent use by staff. Staff members began donning their masks while interacting with the public as a measure of precaution.

On Wednesday, 11 March 2020, a General Staff Meeting was held to sensitise staff on the preventative measures that the NLJ administration would be putting in place. The format was a PowerPoint presentation delivered by the Director User Services and Conservation, who is also the NLJ’s Disaster Coordinator. The topics covered included the situational analysis for Covid-19, how the virus is spread; symptoms of the virus; steps to prevent the illness; and what to do if one becomes ill. Staff was also informed of a risk assessment survey that the NLJ administrators would be undertaking.

After the presentation, staff had a myriad of questions to include, how they would reduce their risks if they utilised public transportation as their primary means of travel? And, if the library had plans to bar entry to persons who were displaying flu-like symptoms? In general, the staff were concerned about their safety and well-being while in the building and what measures would be put in place to mitigate the risk. The CDC had issued guidelines that persons who were not sick did not need to wear masks, and as such, staff were advised that masks were not necessary (CDC, 2019). Staff were reassured coming out of the meeting and expressed gratitude to the management for taking a proactive approach in safeguarding their health and well-being while at work.

By Wednesday evening, on 11 March 2020, the Prime Minister, the Most Honourable Andrew Holness, ON, MP, addressed the nation in a press conference, informing the citizens that, based on the current situation, the government had decided to close all schools. He advised that this was meant to put parents on the alert, so they could formulate alternative solutions when this decision was made. Schools were officially closed by the Ministry of Education, Youth and Information on 13 March 2020 (Public Broadcasting Corporation, 2020b).

On this same day, 13 March 2020, the parent Ministry for the NLJ, the Ministry of Culture, Gender, Entertainment and Sport convened a meeting with all the Disaster Coordinators for the agencies within its purview. In this meeting, the coordinators had to indicate the measures they were each putting in place as it relates to Covid-19. At the end of the session, the Minister announced that with effect from 14 March 2020, all agencies under the ministry’s portfolio would be closed to the public until further notice. A subsequent press release was sent out to all the media houses with the advisory of closure of these facilities. All official communications from the government regarding orders to be put in place would also be facilitated through this disaster group.

As the Disaster Preparedness Team began the implementation of disaster measures, the government of Jamaica mandated the closure of the NLJ to the public on 14 March 2020. Staff were allowed to operate under a flexible work arrangement. All cultural heritage and educational institutions would be closed to the public until further notice. The decision was made in keeping with observations and standards that were being implemented worldwide as the virus was spreading rapidly in some countries. Thus, one of the most robust recommendations that were coming out of governments around the world was to close cultural institutions to the public to safeguard staff, clients and the collection.

In a bid as well to curb the passing of the virus from person to person, on 16 March 2020, the Prime Minister addressed the nation and informed Jamaicans that all non-essential workers in the public and private sector who could work from home should do so to minimise the spread of the virus (Public Broadcasting Corporation of Jamaica, 2020c). As such, this measure would minimise overcrowding in the workplace, as well as minimise the risks for staff that had to take public transportation to work. Additionally, it also presented the opportunity for staff with comorbidities that would represent a risk to them to be able to minimise their need to be in public. Jamaicans were encouraged to stay home to reduce the spread of the virus. This strategy proved fruitful in ‘flattening the curve’, and in local Jamaican patois, the phrase was ‘tan a you yard’ meaning stay at home.

As part of a preventative measure, a risk assessment of the staff was undertaken to determine their level of risk in reporting to work. This risk was based on the distance of commute, pre-existing health conditions such as diabetes, cardiovascular disease, respiratory challenges such as asthma, the mode of transportation and housing facilities such as a multifamily residence. The survey instrument had seven questions with an approximate response time of 7:04 min. Of the 76 staff members on the establishment, 68% responded (35 responded to the online survey and 17 to the paper-based). Fifty-three per cent of the staff classified their level of risk of contracting the virus from medium to high mainly because they use public transportation as their mode of commute. Staff were given the opportunity to make recommendations to management on how the NLJ could assist in managing their risks. The responses ranged from promoting social distancing, restricting access to the public, implementing a work from home strategy, NLJ providing transportation and providing financial assistance for expenses related to Covid-19. The business continuity plan developed by the NLJ has incorporated these measures and ideas that were presented by staff.

The Director, Human Resources Management and Administration and the National Librarian were able to make informed decisions regarding work arrangements based on the risk assessment survey, observation and dialogue with staff. The HR strategy was also in alignment with government strategy (i.e. vulnerable staff to stay home while adhering to Public Sector leave arrangements concerning Covid-19). Four staff members who had pre-existing health conditions and were regarded as high risk were given assigned tasks to work from home. All other staff members were scheduled on the flexible work arrangement where they could ‘work from home’ and were given assigned duties from 18 March to 29 May 2020. The workplace protocol enforced during this period included adhering to social distancing the practice of being 6 feet apart, only two members of staff being scheduled to work in a branch on any given day, the wearing of masks and proper sanitisation while on duty. With these preventative measures, the NLJ to date has no infections or casualties.

## Covid-19 an opportunity or a threat?

The timing of Covid-19 seemed to fit seamlessly with the plans of the NLJ. The NLJ had just begun a significant inventory activity, and staff were given assigned collections to standardise the data. The resilience of the staff is highly commendable as at the beginning of exercise, 14 new laptops were needed. As the NLJ administrators pondered on the possible sources of funding for the laptops, staff volunteered to use their personal laptops and computers to work from home thus saving the library from having to purchase these equipment. With staff being fully engaged, a business continuity plan had to be framed to capture the operational processes of the NLJ. The Managers of each branch were interviewed to ascertain what was needed to continue the branch operations from home. The NLJ subscribes to Microsoft Office 365 that combines premium Office apps with Outlook while offering cloud storage. This software facilitated the hosting of virtual meetings using Microsoft Teams and accessing files remotely from Microsoft One Drive. Communication with staff was facilitated using Microsoft Teams, Whatsapp, email and the office notice board. With the business continuity plan now operational, the NLJ was able to improve the online services, offer new online services such as the online processing of the International Standard Book Number (ISBN). The Covid-19 pandemic period is being viewed more as an opportunity as backlog tasks were addressed.

With the closure of all the schools island wide, the NLJ updated its digital collection to provide more full-text documents and provided linkages to online resources that would be useful to high school students. The NLJ was able to provide these services and preserve the national collection with the acquisition of a hybrid microfilm scanner system that functions as a two-in-one machine because it produces microfilm as well as digital images of paper-based documents; as well as an SMA scanner. The Research and Information Branch waived all costs for research conducted during Covid-19 and have fulfilled requests via email at nljresearch@nlj.gov.jm. The support of the NLJ to students was marketed on the NLJ’s Facebook page. The Jamaica Information Service (Reckord, 2020) and the Ministry of Education, Youth and Information also promoted the NLJ resources to high school students on their websites (Ministry of Education, Youth and Information, 2020).

The NLJ has also compiled a list of trusted sources, to ensure that Jamaicans have access to accurate and authentic information on Covid-19. The web page titled, ‘Get the Facts about the Coronavirus Disease 2019 (COVID-19)’ offers the series of ‘The Disaster Risk Management (Enforcement Measures) Orders’ that was proclaimed and gazetted under ‘The Disaster Risk Management Act, 2015’. The series makes provision for the management and mitigation of disaster, the reduction of risks associated with emergencies and other connected matters (NLJ, 2020b)

The Government of Jamaica has formed an Essential National Health Research Committee, Ministry of Health and Wellness and the NLJ is playing an integral role in the research and data management process. The NLJ team is assisting with framing the Road Map for Jamaica and the acquisition of information on Covid-19 as it relates to Jamaica. The support staff at the NLJ have the responsibility of data entry of the documents sourced. The documents on Covid-19, including grey literature of reports, press releases and other unpublished information are being captured in the research portal of the Virtual Health Library (VHL) https://bvsalud.org/vitrinas/en/post_vitrines/novel_coronavirus/. ‘The Virtual Health Library is a model for the management of information and knowledge, which includes the cooperation and convergence between institutions, systems, networks, and initiatives of producers, intermediaries, and users in the operation of networks of local, national, regional and international information sources favoring open and universal access’ (BIREME, 2020). Jamaicans will be invited to share their personal reflections, photographs, videos, audios and social media on Covid-19, and the NLJ will archive these. The NLJ will be able to preserve the work of the NOW.

## The way forward – Solutions and recommendation to libraries

The Association of Caribbean University, Research and Institutional Libraries, a regional body (Walker, 2020), and international organisations such as Community of Directors of National Libraries (a subgroup of the International Federation of Library Associations (IFLA) (CDNL, 2020) and the Society of American Archivists (Society of American Archivists, 2020) have presented statements or conducted surveys on the impact of Covid-19 on libraries and archives. The study conducted by the CDNL is an interesting read as it gives insights on how 55 national libraries have responded to the Covid-19 outbreak and the impact on their libraries. It should be noted that virtually all national libraries worldwide closed their offices to the public.

**Figure 1.**
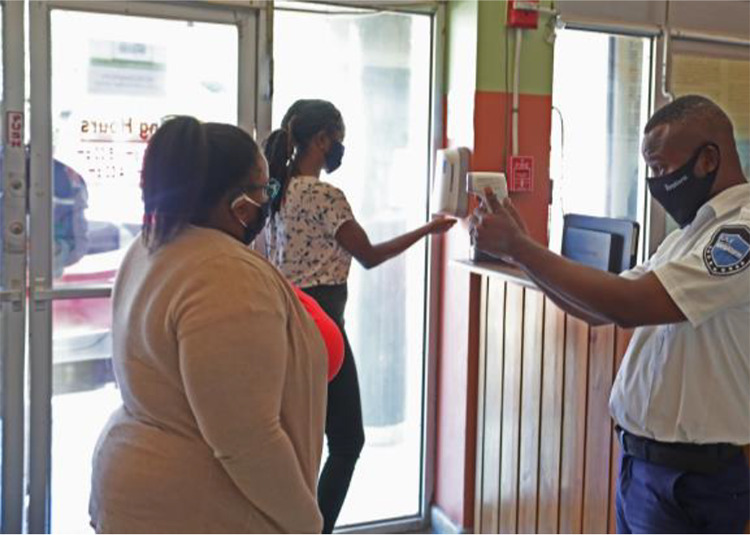
NLJ staff observing protocols.

Investigations were done by the NLJ Research Team regarding how the collections should be handled during the Covid-19 pandemic, however little information was available initially. Colleagues within the library community were contacted and they emphasised the protocols such as washing and sanitising of hands before handling and touching the materials, the wearing of gloves, minimising the number of patrons in the reading areas and observation of the social distancing protocols. Figure 1 below captures the security officer on duty conducting temperature checks, while another staff member uses the hand dispenser to sanitise her hands.

A report conducted by the Canadian Conservation Institute (CCI) and the WHO states ‘COVID-19 could be transmitted by touching contaminated surfaces or objects and by touching one’s eyes, nose, and mouth. Additionally, if an infected person coughs, sneezes, or exhales in the direction of collections or handles objects with contaminated hands, the virus may be transmitted to those who handle the objects afterward. However, it is said that the virus deactivates naturally outside of the human body, so the chances of transmission are said to be low’.

‘The survival of the virus on porous surfaces like cardboard lasted up to 24 hours. However, that was under sort of ideal lab conditions’ (Berendes and Raspberry, 2020). Therefore, best practices dictate the isolation of the collection that are suspected to be contaminated. The CCI recommends isolating the collection for a week to nine days until more testing of SARS-Cov-2 is done (Karsten, 2020).

The article has highlighted the planned preventative measures that the NLJ has implemented to safeguard the staff, clients and national collections against Covid-19. As we continue to grapple with this new virus, which has dramatically impacted our lives and daily habits in every way, Librarians, as information professionals, will have to be aware of new developments that occur to protect and safeguard our cultural heritage. Through trial and error method, our innovative experiments may prove fruitful and will be documented and shared with our networks in the Caribbean region.

The known quotation ‘prevention is better than cure’ applies to the pandemic that has gripped the world. Adhering to the simple protocols of social distancing, washing hands and sanitising surfaces can save lives and the cultural heritage, too, of a country. Understanding the needs of the user plays an integral role in the dynamics of a library, especially during a pandemic. Of vital importance is to continuously update your business continuity plan that covers the security of the collections and staff, as it is estimated that other similar pandemics are possible in the future. The NLJ will keep abreast of trends to offer continued services to its patrons while keeping connected via its online services through shuttered doors.
